# The force effects of two types of polyethylene terephthalate glyc-olmodified clear aligners immersed in artificial saliva

**DOI:** 10.1038/s41598-021-89425-8

**Published:** 2021-05-12

**Authors:** Biao Xiang, Xingxing Wang, Gang Wu, Yichen Xu, Menghan Wang, Yanjing Yang, Qingyu Wang

**Affiliations:** 1 Department of Medicine, Shanghai Smartee Denti-Technology, No. 2305 Zuchongzhi Rd, Pudong New District, Shanghai, 200120 China; 2 Shanghai Stomatological Hospital, Fudan University, No. 356 Beijing East Rd, Huangpu District, Shanghai, 200001 China; 3 Shanghai Brismile Dental Clinic, No. 285 Jianguo West Rd, Xuhui District, Shanghai, 200031 China

**Keywords:** Medical research, Materials science

## Abstract

Numerous factors can influence the force exerted by clear aligners on teeth. This study aimed to investigate the stability of the force delivered by two different material appliances. 90 clear aligners with 2 materials and three different activations were designed and fabricated. Then, a device was employed to measure the force generated by the two types of PET-G material appliances immersed in artificial saliva for 0, 3, 7, 10, 14 days. Scanning electron microscopy was applied to observe the morphologic alterations on the aligner surfaces, respectively. The forces generated by different activation appliance exhibited differently, 0.0 mm < 0.1 mm < 0.2 mm. In addition, increasing the immersion times and the orthodontic force also decreased, but the forces decreased differently. Compared with the forces of conventional PETG appliances with 0.20 mm activation, the modified PETG appliances with the same activation exhibited significantly higher mean force. When comparing the mean force for modified PETG appliances after 10 and 14 days with conventional PETG appliances, the delivery forces exhibited significant differences (*P* < 0.05). The force delivered by both materials decreased obviously following artificial saliva immersion, and the force generated by modified aligners exhibited better stability than conventional aligners.

## Introduction

Clear Aligner Therapy (CAT), first emerging in 1997, was introduced at the American Association of Orthodontist Annual Session (AAO). It was the first technology that incorporated the use of Computer Aided Design/Computer Aided Manufacturing (CAD/CAM) with transparent thermoplastic materials to align the teeth. Development over the last two decades has realized much progress in terms of key materials, digital technology, and clinical application of CAT^1,2^. The earliest clear aligner company in China was built in 2001, and Smartee Denti-Technology, Shanghai, China established in 2004 has become one of the largest CAT companies in China. CAT, which was initially aimed at treating patients with mild-moderate dentition crowding, has nowadays also applied in the treatment of severe crowding and complex malocclusions with improvements in aligner materials, CAT systems, intraoral scanned digital models technology and resin attachments bonded on tooth^3,4^. More and more orthodontists and patients were attracted towards this technology because of comfort, good oral hygiene facilitation and less root resorption rates. Currently, over 6 million patients have been treated with clear aligner technology.

Clear aligners have been utilized extensively in clinical orthodontic treatment; however, it remained problematic to complete treatment of some complex malocclusion patients using CAT. Moreover, CAT still required more improvements when compared to fixed appliances therapy. Fixed appliances exerted relatively constant light forces depending on the deflection of the wire to align teeth until the teeth moved and changed the deflection. Thermoplastic material behaviors exhibited creep and stress relaxation properties over time because of its viscoelastic property, and can commonly led to either high force application on the teeth, causing pain, or too little force, achieving scarce teeth movement^1,5,6^.

The capability of CAT effectiveness was primarily dependent on material property of the aligner. Currently, polyethylene terephthalate glycol-modified (PET-G), polycarbonate (PC), thermoplastic polyurethanes (TPU) and polypropylene materials were widely applied in the manufacturing of aligners. Previous studies have^6^ reported that numerous factors can change the material orthodontic force, including material thickness, removal frequency, wearing time and activation. Clear aligners came with variable thickness, ranging from 0.5 to 1.5 mm, which also affected the force value. Clear aligners with different activation ranging from 0.0 to 0.33 mm can also influence the overall orthodontic force exerted on teeth. Under constant light force, behavior and mechanical properties of clear aligners also underwent changes over time because of its viscoelastic nature property. Studies have further demonstrated that orthodontic forces behaved differently with removal frequency changing^6,7^. The orthodontic force in most previous studies was calculated using virtual three-dimensional finite element analysis^8^, however, the orthodontic force of a simulation model of real conditions that take into account the effect of artificial saliva on clear aligner in our study was measured using a force change detection device.

The constant deflection of clear aligners with viscoelastic properties wearing on tooth could generate relatively constant force to move the teeth until the teeth change the deflection. Numerous studies have investigated the force properties of thermoplastic materials during teeth alignment. Adham Skaik^6^ reported that conventional PETG and modified PETG exhibited a decrease in overall force in two days. Compared with conventional PETG, the force exerted by the modified PETG decreased differently when the clear aligners were removed more frequently, and the modified PETG showed higher stability compared to conventional PETG. This study, however, didn't consider that appliances ware placed on teeth of patients for two weeks when moving and aligning tooth. Few researches have been conducted to tackle this problem, which required further investigation. We hypothesized that immersion in artificial saliva at 37 °C could be used to simulate the oral environment for experimentation.

**Figure 1:**
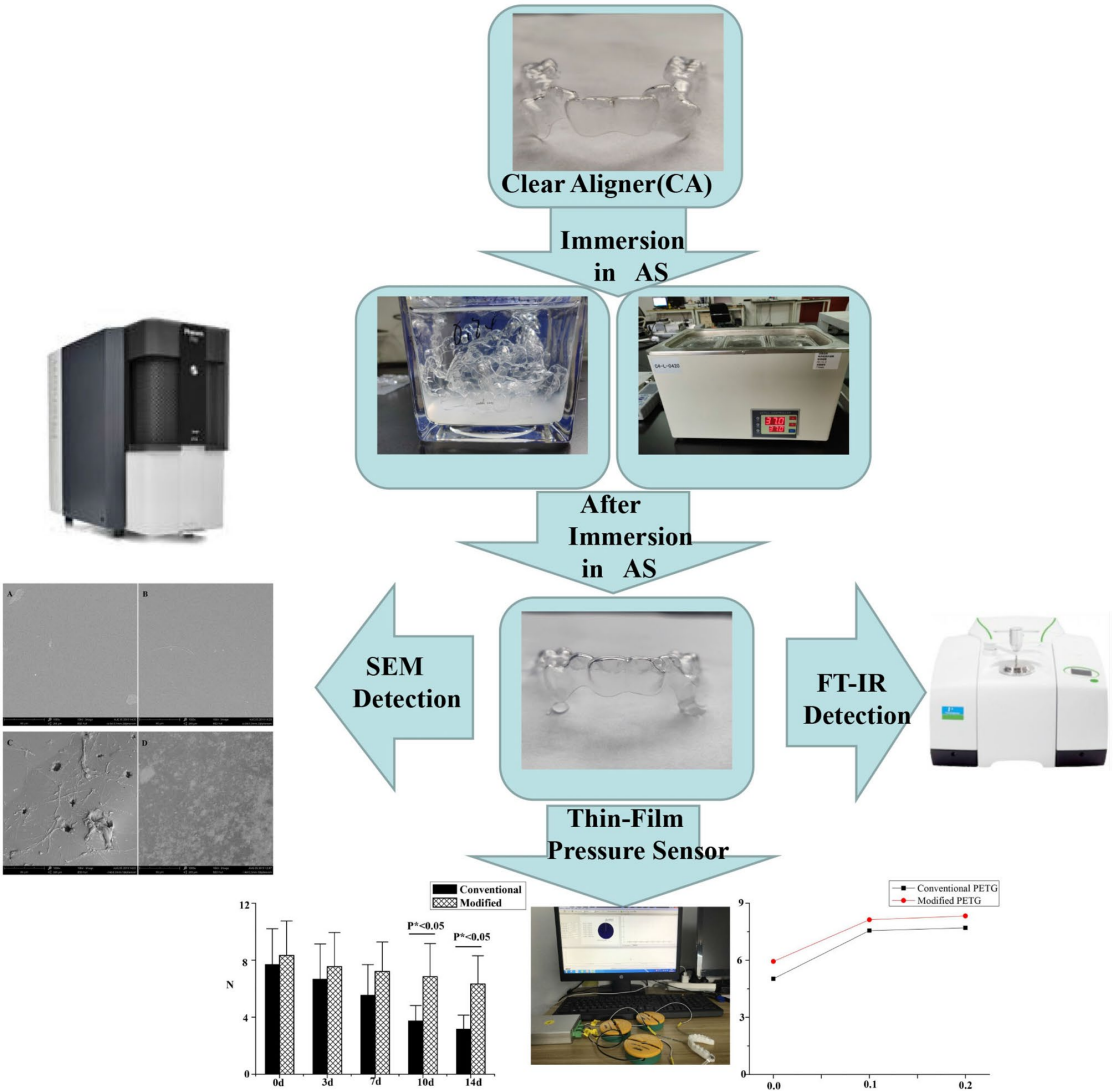
Schematic diagram of clear aligner immersed in AS: fabricating the clear aligners, artificial oral environment, a thin-film pressure sensor detection, scanning electron microscopy detection, and fourier transformation infrared detection.

Previous studies have confirmed that the mechanical properties of clear aligners in simulated intraoral environment had significant difference compared to those clear aligners without in simulated intraoral environment^9^. In our research, we have developed a simulation model of real conditions that took into account the effect artificial saliva on clear aligners. Our aim was to investigate and compare the force stability of conventional PETG and modified PETG immersed in artificial saliva for two weeks with different activations and different immersion time. Finally, the constant light force was measured by a force change detection device. A workflow of the systems material experiment was summarized as shown in Fig. 1.

## Materials and methods

A digital three-dimensional finite element model including the clear aligner, teeth, periodontal ligament, alveolar bone was used to investigate the constant force for conventional solution. In our study, we have developed a simulation model of real conditions that took into account real clear aligner, teeth, periodontal ligament, alveolar bone and saliva to detect the constant force using a thin-film pressure sensor.

### Resin model

The experiment procedures used for this study was reviewed and approved by the Local Ethics Committee of Shanghai Stomatological Hospital, Fudan University, and all methods were performed in accordance with the approved guidelines and regulations. Written informed consents from all the participants were obtained before they entered this experiment. The complete dentition information of patient was obtained. Three-dimensional digital models of the upper dentition were obtained from the same subject using intraoral scanner CS3600 ACCESS (Carestream Health, Shang Hai, China), and saved as STL files. Using the Standard Tessellation Language (STL) files, experimental resin cast was designed and fabricated with the help of the company (Smartee Denti-Technology Co., Ltd, Shanghai, China). Based on the activation between tooth position in the clear aligner and the corresponding tooth position on resin model, the constant deflection of clear aligners with viscoelastic properties wearing on tooth could generate relatively constant force to move the teeth until the teeth move to the position of tooth in clear aligners. According to varying teeth movement, the resin model was made using three different activations, including 0.0 mm, 0.1 mm and 0.2 mm.

### Clear aligner

**Figure 2:**
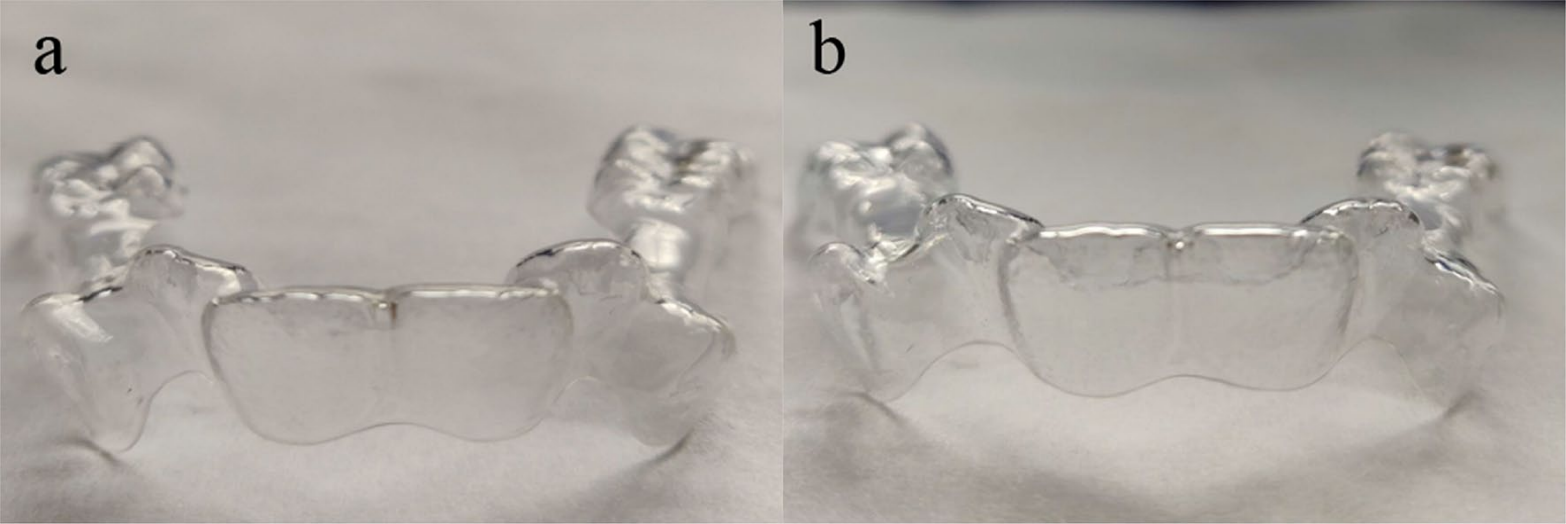
Picture of the aligner. (**a**) Conventional aligner; (**b**) modified aligner.

A series of clear aligners with three activations, 0.0 mm, 0.1 mm and 0.2 mm at the maxillary central incisor, canine, second premolar and first permanent molar were designed and constructed by clear aligner Co., Ltd. The clear aligners were structured with two different materials, the first material being conventional PETG with lower modulus elasticity and abrasion resistance; while a modified material with higher modulus elasticity and greater abrasion resistance was chosen as the second material. Both the materials were 0.75 mm thick layer, and primarily composed of modified-polyethylene terephthalate glycol, and the two different material aligners were shown in Fig. 2.

### Artificial oral environment

**Figure 3:**
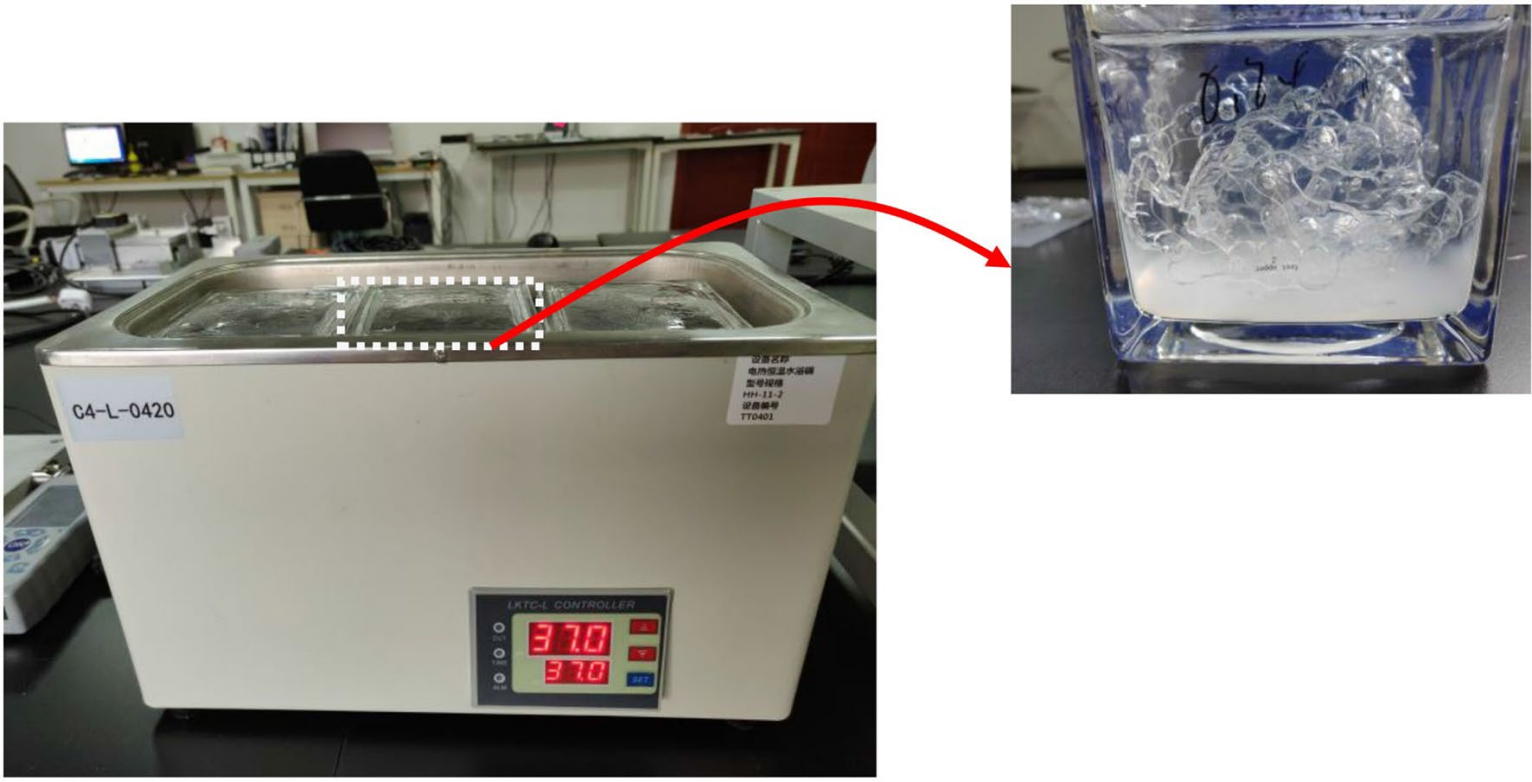
Schematic of imitating the oral environment: clear aligner immersed in AS at 37 °C for 3 days, 7 days, 10 days, 14 days, heating setup with water-bath thermostat, and the AS was refreshed every day.

The artificial saliva was consist of NaCl, KCl, Cacl_2_·2H_2_O, KH_2_PO_4_, Na_2_HPO_4_·12H_2_O, KSCN, NaHCO_3_ and C_6_H_8_O_7_. Clear aligners (n = 90) were immersed in Artificial Saliva (AS) at 37 °C to simulate oral environment, and 15 clear aligners were immersed in 200 mL artificial saliva, as depicted in Fig. 3, and were subsequently divided into the five following groups: control group (AS immersion for 0 day), 3 days group (AS immersion for 3 days), 7 days group (AS immersion for 7 days), 10 days group (AS immersion for 10 days), 14 days group (AS immersion for 14 days). AS was refreshed every day in each group. After the respective time periods were met, the clear aligners were taken out and washed with distilled water.

### A thin-film pressure sensor detection

**Figure 4:**
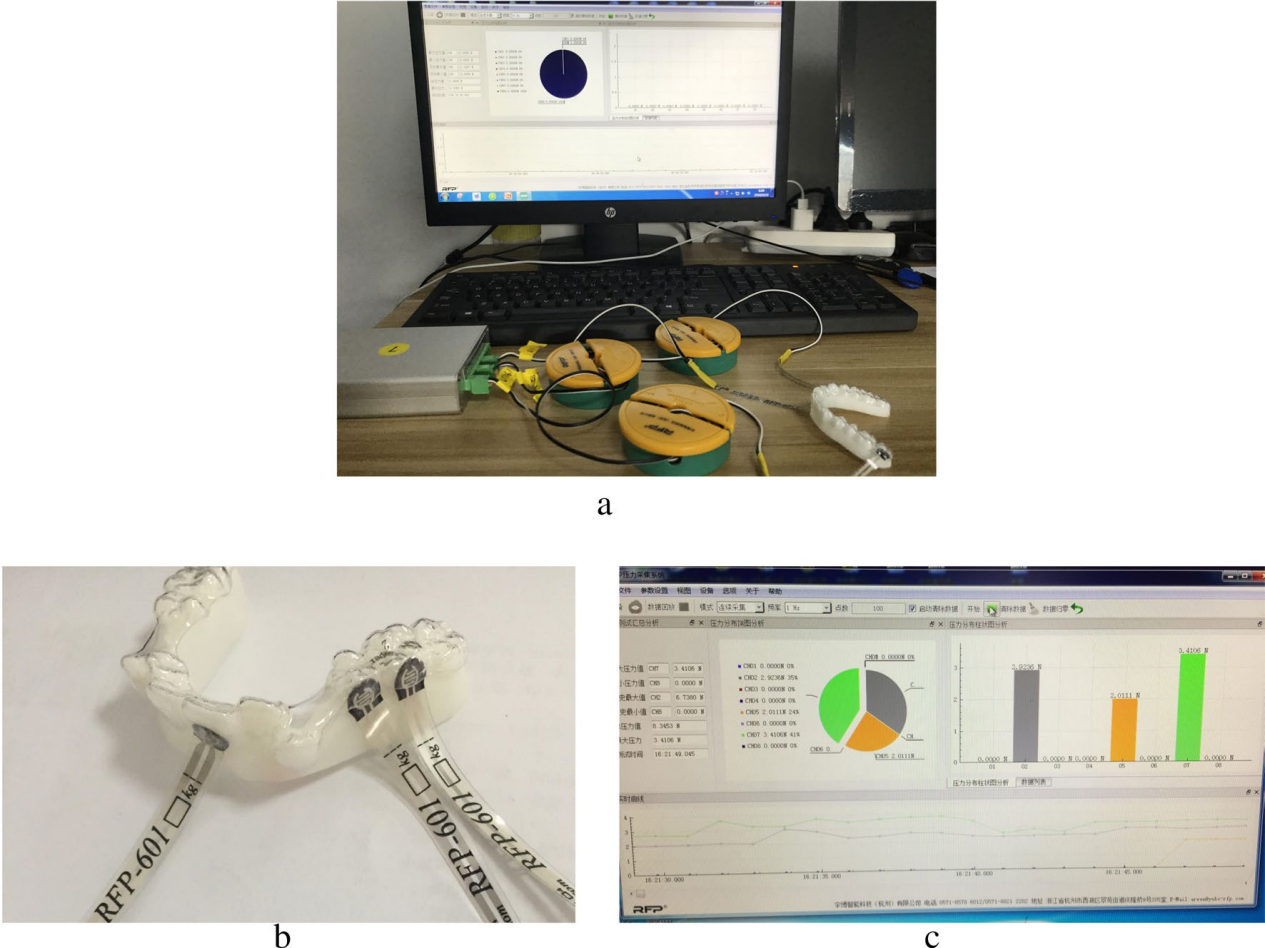
(**a**) Shows the setup of the thin-film pressure sensor measuring the constant force; (**b**) shows 0.2-mm thin ultrathin flexible printed circuit sensors, which were very thin and flexible to adjust to contact surface between the clear aligners and resin model, were constructed, and the feature of the resin model was specially designed and minimized to provide space for the sensor. The sensors were placed on the resin model, and then the aligners were also placed on the resin model. The sensor output data was connected to the circuit board, which was primarily for signal acquisition. (**c**) Shows system of tested forces, of which the export signal of circuit board was transferred to a computer to visualize the orthodontic force value.

A thin-film pressure sensor detection system was established in order to measure the force stability, which comprised of a thin-film pressure sensor, a signal acquisition circuit board, a software visually measuring the pressure date, a computer, and the sensor’ structure was shown in Fig. 4. Regarding the sensor thickness as the primary factor when measuring orthodontic force, the sensor thickness was specifically designed to provide space according to the feature of resin model; subsequently, 0.2 mm thick sensors, the thinnest sensors of their kind were designed and constructed by Yu Bo Intelligent Technology Co., Ltd (Hangzhou, China). A calibration process was carried out to reduce the system error. The sensor was fixed on a standard flat plate and a series of known force from 0 to 5 N at an interval of 0.5 N was tested by the sensor and record the measured force value, and all data were repeat 3 time and got the average value. The force differences between the known force and the measured force were limited to less than 0.2 N, which was acceptable. The positions of measuring force points were at the maxillary central incisor, second premolar and first permanent molar.

### Scanning electron microscopy detection

The clear aligners, which were immersed in AS, were prone to material surface aging, peeling and pore formation, and Scanning Electron Microscopy (SEM) detection was employed to explore the differences in aligners surface morphology. Specific area specimens of the maxillary central incisor, canine, second premolar and first permanent molar were cut and measured by SEM at 1000 ×, 5000 × magnification.

### Fourier transformation infrared detection

Based on the transmittance measurements, FT-IR spectroscopy was applied to examine the chemical group of the surface morphology of the aligners immersed in AS in attenuated total reflectance mode. Before and after 14 days of AS immersion, maxillary first molar part of the aligners was cut and extracted (n = 3). The specimens were washed in distilled water for 5 min and dried with tissue paper. Spectra of the two type of material aligners were measured over the range of 600–4000 cm^−1^ with an FT-IR spectrometer, and FT-IR spectra were analyzed by software.

### Ethics declarations

The experiment was approved by the local ethics committee of the Smartee Denti-Technology and written informed consent were obtained from all the participants.

### Approval for human experiments

The experiment was approved by the patient.

## Results

### Effects of AS on force generated by two types of material aligners

**Figure 5:**
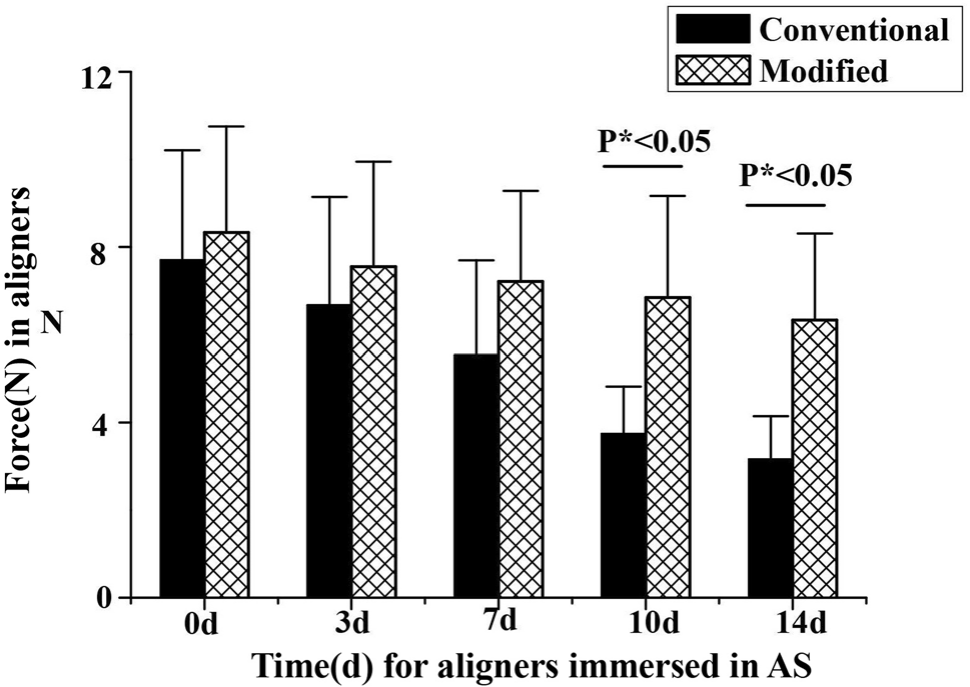
Shows the mean forces of conventional and modified clear aligners respectively immersed in AS for 0 day, 3 days, 7 days, 10 days and 14 days, and the force values were presented as means ± standard deviation. P* < 0.05 versus conventional groups, n = 3.

Orthodontic force value was measured to evaluate the stability of two different material aligners immersed in AS. As shown in Fig. 5, the force values were lower in all clear aligners groups immersed in AS (immersion for 3 days, 7 days, 10 days and 14 days) compared to those in the control group (immersion for 0 day). In addition, when compared to the conventional PETG aligner group, the force values of modified PETG group were higher, while the force values were significantly elevated following AS immersion for extended periods (10 days, 14 days). These findings demonstrated that the force generated by modified PETG decreased relatively slower than that in conventional PETG induced by AS.

### Effects of different activation on force generated by two types of material aligners

**Figure 6:**
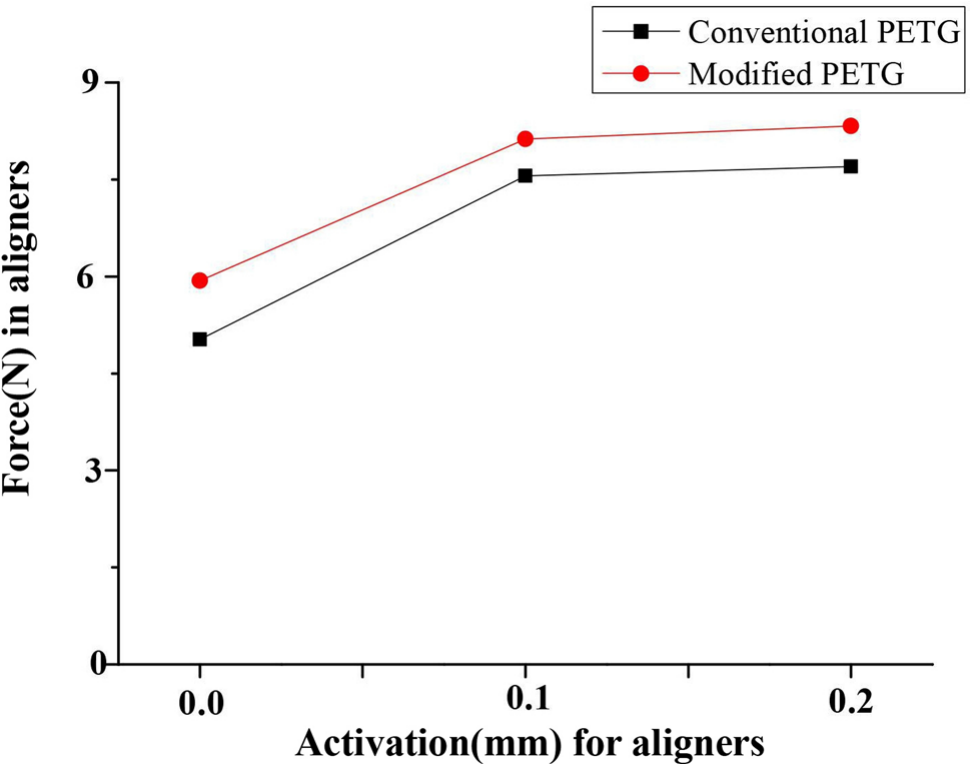
Shows the mean forces of conventional and modified clear aligners with activation of 0.0 mm, 0.1 mm and 0.2 mm, respectively, and the force values were presented as means ± standard deviation. *P < 0.05 versus conventional groups, n = 3.

Force values of two material properties aligners with three different activation (0.0 mm, 0.1 mm and 0.2 mm) were measured to investigate the force patterns exerted by varying activation aligners on teeth. As depicted in Fig. 6, the force values in 0.1 mm and 0.2 mm groups were higher compared to the 0.0 mm group, with the orthodontic force being the highest in the 0.2 mm group. In addition, force values were found to be much higher in the modified PETG group relative to the conventional PETG aligner group.

### Effects of AS on aligner material surface morphology

**Figure 7:**
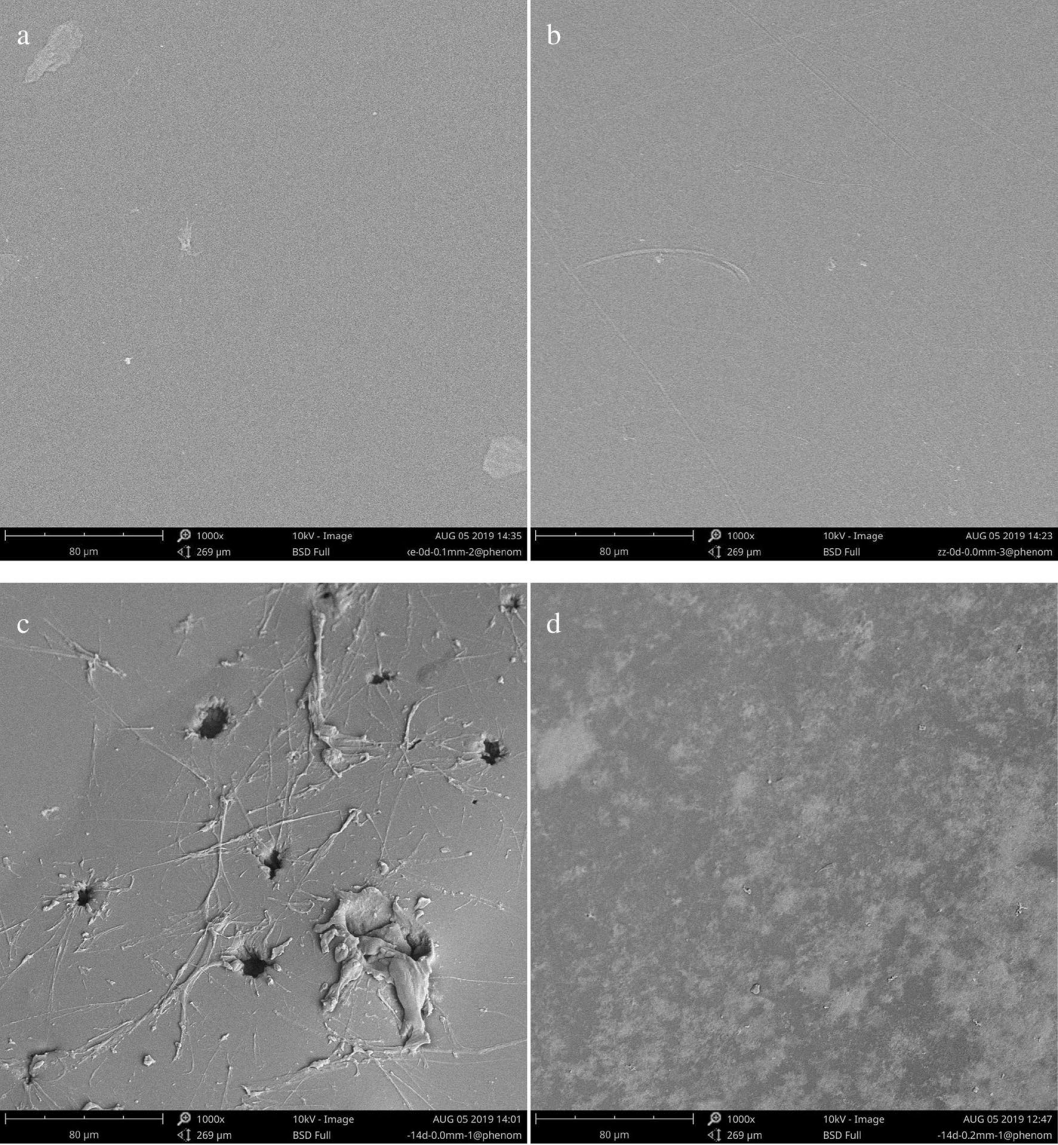
The aligner surface morphology immersed in AS examined by SEM. The aligners were administrated respectively with AS for 0 and 14 days. (**a**,**c**) conventional PETG aligners, (**b**,**d**) modified PETG aligners; (**a**,**b**) before immersion; (**c**,**d**) after immersion.

Additionally, SEM was applied to study the aligner material surface morphology and the results were shown in Fig. 7a–d. Compared with control group (immersion for 0 day), AS immersion for 14 days group tended to be rougher and exhibited more severe peeling and increased pore formation. Moreover, compared with the modified aligner material, the surface morphology in the conventional aligner material group presented with significantly more peeling following AS immersion for 14 days. These findings demonstrated that AS immersion caused damage to the surface morphology of both the material aligners, whereas the modified aligner material conferred better protection on the material surface.

### Effects of AS on aligner material spectroscopy

**Figure 8:**
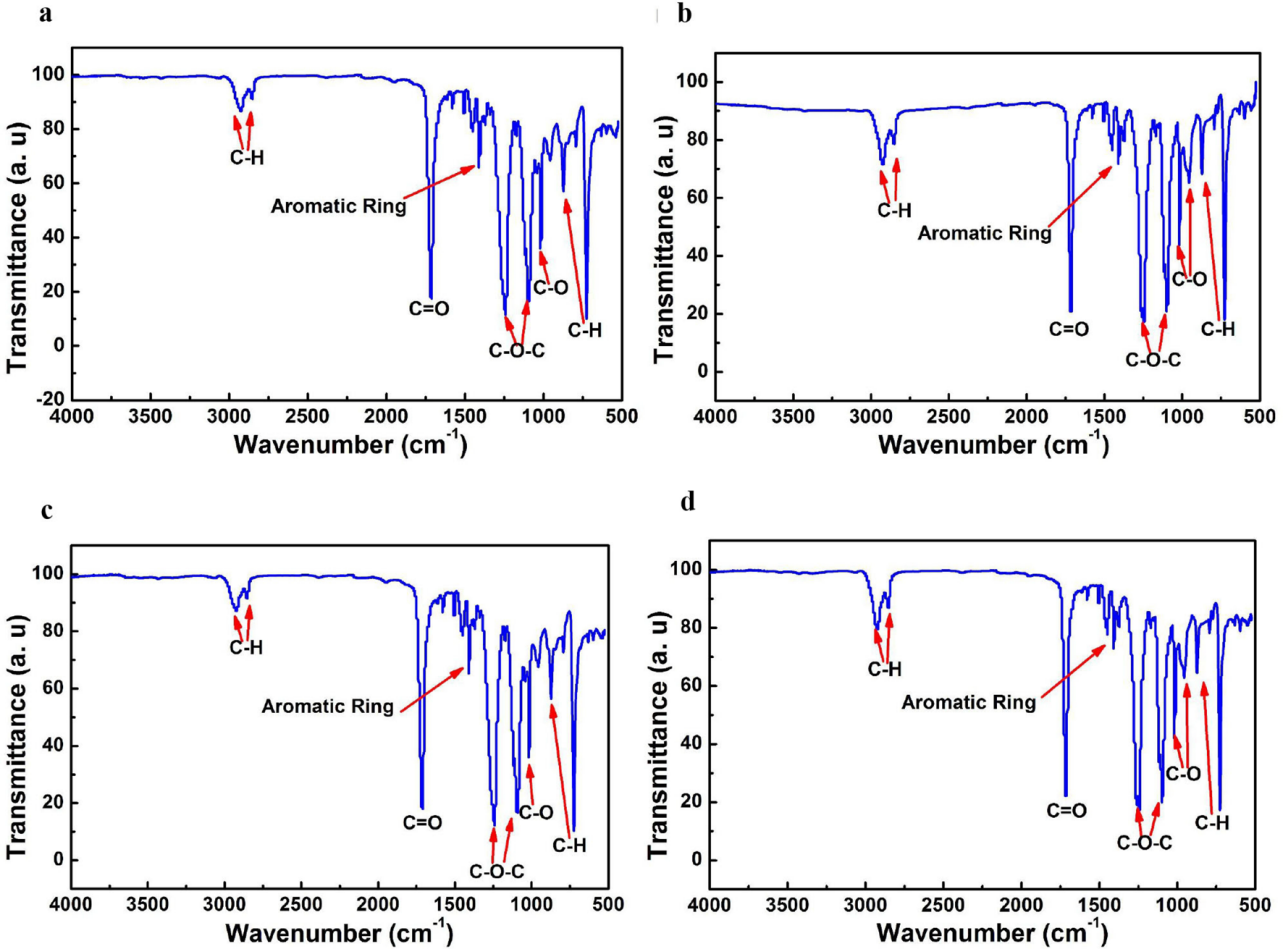
ATR-FTIR spectra of the aligner materials with different immersion time, and the aligners were administrated with AS for 0 and 14 days, respectively. (**a**,**c**) Conventional PETG aligners, (**b**,**d**) modified PETG aligners; (**a**,**b**) before immersion; (**c**,**d**) after immersion.

The chemical groups were measured in the aligner surfaces using ATR-FTIR, and the spectra of the aligners surface materials with 0 day and 14 days immersion time were shown in Fig. 8. The bands at (a, b, c and d) 724 and 873 cm^−1^ exhibited a characteristic peak of the two material aligners according to the C-H bending of the single substituted phenyl ring. The band at (a, b, c and d) 1713 cm^−1^ was the characteristic peak of aligners surface material with C=O stretching of ester groups, and the bands at (a, b, c and d) 1241 cm^−1^ and 1094 cm^−1^ were identified as the characteristic peak of O=C–O stretching. The characteristic peak at 1408 cm^−1^ was identified as the aromatic ring; considering the bands at 2922 cm^−1^ and 2851 cm^−1^, the characteristic peak was identified as C–H bond stretching vibration. Compared with the bands of (a, c) at 2922 cm^−1^ and 2851 cm^−1^, the bands of (b, d) were higher and sharper.

## Discussion

The clear aligner, which could exert constant light force to move the teeth, completed treatment based on wearing a series of aligners made of transparent thermoplastic materials. Manufacturers have the choice of three thermoplastic materials in the production of clear aligners, namely TPU, TPC, and PETG. Recent studies have indicated that material thickness, time and activation were among the various factors capable of affecting the orthodontic force value exerted on teeth. Barbagallo et al. documented a mean force magnitude of 5.12 N with 0.5 mm activation utilizing a 0.8 mm thick material, whereas, Kohda et al. obtained a mean force magnitude of 1.65 N with 0.5 mm activation using a 0.8 mm thick material. Therefore, the thickness and the activation had visible influence on the orthodontic force value^10–12^. More intriguingly, Adham Skaik et al.^6^ investigated the effects of wearing time and activation amounts on the force of two PETG material aligners, and uncovered that the force value of both materials decreased with time and increased with increasing activation, however failed to simulate the intraoral environment for pragmatic results. However, earlier studies have investigated the potential effect between constant force on tooth movement and intermittent force on tooth movement. The results suggested that intermittent force in periodontal ligament (PDL) cells had less damage than that constant force in PDL cells had. In order to further investigate the effect of constant force on tooth movement^13,14^. In the current study, we established an intraoral environment to investigate the effects of immersion time and activation on orthodontic force value.

Several difficulties were faced in the measurement of orthodontic force on the contacts surface between the aligners and models, which can easily lead to measurement error owing to the fact that the contact surfaces were curved instead of flat^6,15^. In the current study, a thin-film pressure sensor, which was simplistic, high-precision and low-cost, was specifically designed and utilized to examine the force values. The accuracy of thin-film pressure sensors has been recognized in the measurement of force of the contact surface between model and aligner, and the force measurement errors between the simulation and experimental models were less than or equal to 0.2 N, of which measurements value was accepted. In addition, complicated contact surface between aligners and resin models can also frequently lead to sensor weight imbalance, causing measurement error. To tackle this, we designed and manufactured an adequate thin and flexible sensor, which was 0.2-mm in thickness, to adjust to the measurement area, and employed a pressure-film approach to measure the force exerted by thermoplastic appliance on the first maxillary molars in vivo which has been investigated in some previous studies^16,17^.

Currently, clear aligners were available with different activation, ranging from 0.0 to 0.33 mm. Numerous studies have highlighted that the amount of activation can modulate the orthodontic force; however, such values remained to be investigated, and the continuous light orthodontic force was delivered to the target tooth for effective tooth movement^18,19^. Interestingly, different activation-based approaches have also been discovered to affect the clear orthodontic force value, but, the detail mechanism of different-activation affecting the orthodontic force value required more research^20^. For fixed orthodontic therapy, the amount of tooth movement was primarily dependent on orthodontic clinical experience. The orthodontic force on the tooth was either too much, causing root resorption, or too small, obtaining little movement effect. One particular study explored the value of clear aligner therapy using the force value of 0.0 mm, 0.1 mm and 0.2 mm three different tooth movements, and found that the orthodontic force value changed with the amount of activation as follows: the force of 0.0 mm activation < the force of 0.1 mm activation < the force of 0.2 mm activation, and provided the foundation for different activation aligners therapy^6,21^. In the current study, we examined the effect of three different activations, 0.0 mm, 0.1 mm and 0.2 mm on the orthodontic force properties using AS immersion, and our findings were in line with those reported in Adham Skaikpr’s papers.

Aligners being inside oral cavity were vulnerable to water adsorption, which can commonly lead to unstable mechanical properties and degradation of aligner material, consequently failing to provide adequate force to control tooth movement. The mechanical properties of aligners have also been associated with wearing time, and studies have suggested further that PETG, TPC and TPU, all different aligner materials exhibited different water adsorption rates in simulated intraoral environment. Attachment and penetration of water molecules into the material surface has also been shown to cause adverse effects on the functioning of the aligner^7,22^. Furthermore, studies have also identified that the duration of wearing time can significantly alter the surface morphology and mechanical properties of aligners, causing evident changes in overall functioning. Based on previous papers and the aligners wearing in the oral cavity time (7 days or 14 days), in the current study, we utilized different immersion times (3 days, 7 days, 10 days and 14 days) to investigate the detailed change mechanism of aligner material surface morphology and mechanical properties, and the result revealed that intraoral environment simulation caused visible variations in material surface morphology and mechanical properties in both the aligner materials.

Furthermore, the change in the orthodontic force among different aligners have been reported by some previous papers, Adham Skaik found that the orthodontic force with 2 material properties and (0.0 mm, 0.1 mm and 0.2 mm) three different activations at the maxillary permanent first molar, maxillary second premolar and maxillary central incisor exhibited similar decreases over time. In the current study, a 3D digital model of maxillary the first premolar extraction case was utilized to investigate the orthodontic force. To explain the detail mechanism of clear aligner closing tooth extraction gap, the force value of the maxillary canine also was measured; therefore, we calculated the orthodontic force of the maxillary canine, incisor, second premolar and first permanent molar^23,24^. Both the aligner materials exhibited decreases in orthodontic force after in vitro simulation (3 days, 7 days, 10 days and 14 days). However, it was notable that the orthodontic force generated by the modified clear aligners was more stable compared to those in conventional aligners. Similarly, the modified aligners exhibited significantly higher orthodontic force values following AS immersion for 10 and 14 days compared to the conventional aligners. The clear aligner, which was viscous, elastic and even intermediate properties material, exerted constant orthodontic light force depending on the deflection of the thermoplastic material. The aligners properties also changed over time even though the tooth has not moved. Under orthodontic force, the deflection of thermoplastic material increases over time, while aligner material load decreases with the constant deflection because of its creep and stress relaxation; therefore, the thermoplastic material, the wearing time and amount of activation affected the force value^6,22,25^.

After the discovery that mean force generated by modified aligners was higher than that generated by conventional aligners, the clear aligners were immersed in AS to study variations in aligner surface morphology. Furthermore, the thermoplastic material surface morphology usually has been associated with deflection of the clear aligners, and the continuous orthodontic light force was exerted by deflection of the aligners. Some studies have investigated the surface morphology variations caused by immersion in coffee, black tea, red wine, and 75% ethanol solution, and aligners did exhibit surface morphology changes, but these solutions were unable of truly simulating the oral environment, which might not reflect the realistic conditions of aligners placed on tooth^7,26,27^. Hence, we chose to immerse the clear aligners in AS at 37 °C, and measured the forces exerted on teeth and surface changes. SEM resulted from our study illustrated that the conventional-based material presented with rougher surface morphology and more peeling. Moreover, modified aligners generated higher orthodontic force values after AS immersion for 14 days compared to the conventional aligners. Both the materials investigated in the current study were composed of PETG polymers, and the aligners surface group contained a hydrophobic group; however, the modified PETG aligners displayed better stability, which might be associated with the material surface group and penetration rate. Some previous papers have reported that three types of polymers-based materials exhibited different water adsorption rates, and water penetration into material surface led to aligner surface morphology change. Water adsorption rates which usually depended on hydrophilic or hydrophobic polarity of the thermoplastic material surface, might influence the mechanical properties of aligner.

Moreover, water adsorption rates have been associated with surface groups, and the aligner groups were analyzed using ATR-FTIR. Within the aligners surface material spectra, the hydrophilic groups were prone to hydrogen bond formation, which interacted with water penetration^28,29^. The peak shape changed less before and after immersion of clear aligners in AS. Both conventional aligners and modified aligners contained C=O stretching at 1713 cm^−1^, O=C–O stretching at 1241 cm^−1^ and 1094 cm^−1^. Compared with conventional aligners spectra wavenumbers, the strongest bands showed no shifts. However, the bands of (a, c) at 2922 cm^−1^ and 2851 cm^−1^ of modified aligners were higher and sharper, and the C–H bond stretching vibration was the hydrophobic group, which could decrease the water adsorption rates and increase the material stability. These results showed that C–H at 2922 cm^−1^ and 2851 cm^−1^ may increase the material aligner surface stability.

## Conclusion

The force delivered by both PETG thermoplastic materials decreased obviously following artificial saliva immersion, and the mechanical force generated by modified aligners, surface morphology and structure exhibited better stability than conventional aligners. Therefore, the modified material could be a promising material for orthodontist and patients owing to its ability of appropriate delivery force stability.

## Data Availability

The datasets in this manuscript are available from the corresponding author according to appropriate request.

## Abbreviations

PETG: Polyethylene terephthalate glycol-modified
SEM: Scanning electron microscopy
CAT: Clear aligner therapy
CAD/CAM: Computer aided design/computer aided manufacturing
PC: Polycarbonate
TPU: Thermoplastic polyurethanes
FEA: Finite element analysis
AS: Artificial saliva
